# Feasibility of the heaviness perception test as an assessment of interoception

**DOI:** 10.1186/s13030-025-00343-x

**Published:** 2025-10-31

**Authors:** Daisuke Fujimoto, Masahito Sakakibara

**Affiliations:** 1 Aichi Medical College for Physical and Occupational Therapy, 519 Ichiba, Kiyosu City, Aichi Prefecture 452-0931 Japan; 2 Department of Psychology, Aichi Gakuin University, 12 Araiie, Iwasaki-Cho, Nisshin City, Aichi Prefecture 470-0195 Japan

**Keywords:** Interoception, Heaviness perception test, Body Perception Questionnaire, Shitsu-Taikan-Sho-Scale, Toronto Alexithymia Scale, Heartbeat counting task

## Abstract

**Background:**

Interoception refers to the multisensory integration and perception of the body’s internal state within the central nervous system, which involves learning, memory, emotions, and experiences. Interoceptive dysfunction has been associated with alexithymia and alexisomia. Despite growing academic interest in interoception, standardized evaluation methods have yet to be established. The widely used Heartbeat Counting Task (HCT), a representative method for assessing interoceptive accuracy, has limitations owing to the potential influence of knowledge of heart rate, time perception, and tactile sensations. Therefore, more reliable assessment methods are needed. This study focused on the feasibility of the heaviness perception test as a method for assessing interoceptive accuracy and investigates its relationship with other interoceptive indices.

**Methods:**

A total of 41 healthy volunteers (19 female; mean age 19.1 ± 0.8 years) participated in the study. The heaviness perception test was conducted using an approach similar to the method of adjustment applied to psychophysical measurements, and the absolute error scores were calculated. Other interoceptive indices investigated in this study include the HCT, Body Perception Questionnaire-Body Awareness Very Short Form (BPQ-VSF), the 20-item Toronto Alexithymia Scale (TAS-20), and Shitsu-Taikan-Sho-Scale (STSS) for alexisomia.

**Results:**

Interoception accuracy assessed using the heaviness perception test showed a significant positive correlation with the BPQ-VSF score (*r* = .504, *p* < .01) and a negative correlation with the TAS-20 and STSS scores (TAS-20: *r* = –.342, *p* < .05; STSS: *r* = –.353, *p* < .05). However, there was no correlation between the heaviness perception test score and the absolute error score on the HCT.

**Conclusions:**

The results suggest that the heaviness perception test is a feasible and useful method for assessing interoceptive accuracy and that it may be useful as an evaluation tool.

## Background

Interoception refers to the integration and perception of the body’s state through multisensory signals within the central nervous system, including learning, memory, emotions, and experiences [1]. The internal body state involves sensory signals, such as pain, temperature, itching, muscular and visceral sensations, vasomotor sensations, and hunger [2]. These sensory signals ascend via lamina I in the spinal dorsal horn and the trigeminal, glossopharyngeal, and vagal tracts to the nucleus tractus solitarius and the parabrachial nucleus [3]. The signals then project upward to the periaqueductal gray area, hypothalamus, and thalamus, further ascending to reach the limbic, anterior cingulate, and insular cortices, where various types of information is integrated [3]. Interoception is not limited to homeostasis; it also contributes to human life skills through emotion formation [4], decision-making [5], pain [6], and the establishment of a sense of self [4]. Some studies have examined interoception from a developmental perspective. Maister et al. [7] showed that five-month-old infants can perceive interoception. They presented videos of animated characters that moved either in or out of sync with the infants’ heartbeats, observing a significant difference in viewing time. Koch and Pollatos [8] reported that children’s interoception is positively correlated with their emotional abilities.

Several studies in the field of psychosomatic medicine have focused on the relationship between personality traits (e.g., alexithymia and alexisomia) and interoception in psychosomatic disorders. Alexithymia, a state of poor awareness of one’s emotions [9], and alexisomia, a state of poor awareness of one’s own body [10], are associated with interoceptive dysfunction [11]. The 20-item Toronto Alexithymia Scale (TAS-20) [12] is used internationally to evaluate alexithymia; its score is negatively correlated with interoception [13]. The Shitsu-Taikan-Sho Scale (STSS) [10] was developed to evaluate alexisomia. Although the correlation between the STSS score and interoception was not examined, a study associated interoceptive dysfunction, assessed using a self-report measure, with alexisomia [14].

Increasing interest in interoception has also increased the need for its appropriate evaluation [13]. Khalsa et al. [15] distinguished between factors such as sensibility and accuracy (attention) when evaluating interoception. The questionnaire is mainly used to evaluate interoceptive sensibility (the degree to which one is aware of interoception) [16, 17]. For example, the Body Perception Questionnaire-Body Awareness (BPQ-BA) was originally a self-report measure of awareness of bodily sensations [18], but it is also used as an index to evaluate interoception sensibility [15, 19–23]. Furthermore, the BPQ-BA score is positively correlated with insular cortex volume [24]. Interoception accuracy has been evaluated via paradigms to objectively quantify individual differences in behavioral performance (an attention task focused on the body) [13, 25]. For example, the Heartbeat Counting Task (HCT) [26] has been used to assess interoception accuracy based on cardiac perception [27]. Individuals are asked to report their heart rate over a specific period of time and, at the same time, their actual heart rates are measured by a device. The degree of discrepancy between the reported and actual rates is then calculated. However, there are several problems with this method: 1) the heartbeat can be perceived through tactile vibrations of the chest wall; 2) individuals may have differing levels of knowledge of heart rate, and 3) individuals may have varying abilities to estimate time [27].

In this study, we investigated the heaviness perception test as a simple alternative to the HCT for evaluating interoception accuracy. Heaviness perception is an individual’s subjective sensation of “weight.” It is mediated by physical weight as well as by proprioception, vision, experience, and the fingers holding an object [28, 29]. Interoception was originally a term referring to visceral sensory information [30], but a broader concept has since been proposed to include the muscle tension state and proprioceptive information [13, 15, 31, 32]. For example, in 2016, leading researchers in interoception gathered for an international conference, the first Interoception Summit, where physiological processes potentially involved in interoception were systematically reviewed. "Muscle tension" was explicitly included as one of these processes [15]. Afferent information on muscle tension and proprioception is essential for the perception and cognition of heaviness; hence, the heaviness perception test can be used to evaluate interoception.

Some studies have demonstrated that heaviness perception can be used to evaluate interoception accuracy [13, 33]. Murphy et al. [13] conducted the heaviness perception test to evaluate interoception accuracy and examined its relationship with the TAS-20 measure of alexithymia. They reported a significant negative correlation. In their experiment, the participants were given a bucket containing rice of a certain weight (e.g., 350, 510, or 780 g). This was then replaced with an empty bucket. The experimenter began to pour rice into this empty bucket, and the participants were instructed to signal when they felt that the weight of the bucket was the same as that of the initial bucket filled with rice. The difference between these weights was then evaluated as an index of interoceptive accuracy. Their findings suggest that interoception assessed using the heaviness perception test may be related to the degree of alexithymia. Moreover, the TAS-20, which evaluates the degree of alexithymia, is composed of concepts such as difficulty identifying feelings (DIF), difficulty describing feelings to others (DDF), and externally oriented thinking (EOT) [9]. It is thought that alexithymia is apparent with any of these impairments. However, the relationships between these components and interoception have not been examined in detail. The STSS, which is used to evaluate alexisomia, includes the concepts of difficulty identifying bodily feelings (DIB), overadaptation (OA), and lack of health management based on bodily feelings (LHM) [10], but the relationship between these factors and interoception has also not been examined.

Therefore, this study was conducted with the heaviness perception test (i.e., investigated interoception accuracy) to comprehensively examine whether it correlated with the BPQ-BA (i.e., interoception sensibility), TAS-20 (total score; DIF, DDF, and EOT), which evaluates alexithymia, and STSS (total score; DIB, OA, and LHM), which evaluates alexisomia. We also examined whether the HCT, which evaluates interoception accuracy, is correlated with these scales. This study used the same procedure as that used by Murphy et al. [13] but replaced the contents of the bucket with water instead of rice. We also used an automatic water dispenser to pour water into the empty bucket at a constant speed.

## Methods

### Participants

A total of 41 healthy volunteers (19 female; mean age 19.1 ± 0.8 years; body mass index 21.0 ± 2.4) participated in this study. None of the participants reported any musculoskeletal of the upper limbs, respiratory, or cardiovascular disorders, nor were they taking any medications on the day of the experiment. All the participants provided written informed consent for inclusion in the study. This study was approved by the Ethics Committee of the Aichi Medical College for Physical and Occupational Therapy (No. 24015).

### Procedure

The participants completed a demographic information sheet (sex, age, body mass index, medical history, and medication use) and a handedness questionnaire [34], followed by self-report questionnaires (BPQ-BA, TAS-20, and STSS). Subsequently, the participants completed the HCT and heaviness perception test. The order of the tests was counterbalanced among participants. After completing these procedures, a cold-pressor test [35] was conducted to address the other research objectives. Electrocardiograms were recorded using a portable device (WHS-1; Union Tool).

### Heaviness perception test

**Fig. 1:**
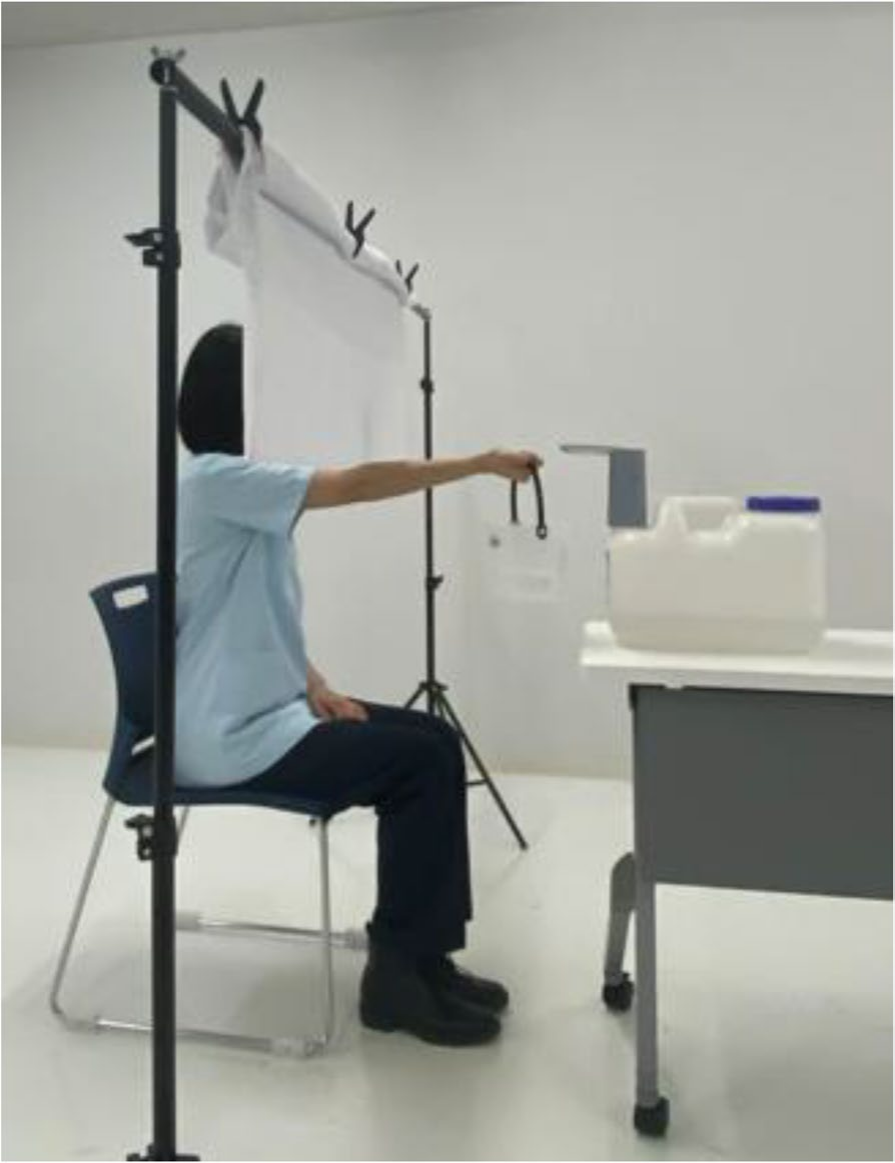
Overview of the heaviness perception test. To prevent visual estimation, a screen blocked the view of the participants hands and the buckets during the test. Participants were seated with their dominant arm extended anteriorly at shoulder height (90° shoulder flexion) and the palm facing upward. After grasping a bucket of randomly selected weight, it was replaced with an empty one, and water was poured at a constant rate using a water dispenser

The heaviness perception test was conducted using the method of adjustment described in the psychophysical paradigms systematized by Fechner, which includes constant stimuli, limits, and adjustments [13, 36]. The procedure used in this study was adapted from Murphy et al. [13] (Fig. 1). Specifically, buckets (capacity 1.5 L, 102.5 g) were filled with water to weights of 350, 510, or 780 g. The test was conducted in three consecutive trials with randomly selected weights and a two-minute interval between trials. To prevent visual estimation, a screen blocked the view of the participant’s hands and the buckets during the test. Participants were seated with their dominant arm extended anteriorly at shoulder height (90° shoulder flexion) and the palm facing upward. The experimenter weighed the bucket, the weight was recorded, and the bucket was placed on the participant’s metacarpophalangeal joints for three seconds, after which the participant grasped the bucket. After replacing the bucket with an empty one, water was poured into this empty bucket at a constant rate (20.0 ± 0.4 g/s) using a water dispenser (B0CL7XBZSX, Broleo). The participants were instructed to signal to the experimenter to stop when the bucket felt equal in weight to the previously held bucket. When the participant said “Stop,” the experimenter immediately terminated the water flow. Participants were not informed of the speed at which the water was poured into the bucket. After each trial, the experimenter weighed the bucket and recorded its weight.

The participant’s score was calculated as “1 – absolute error scores ([actual weight – participant’s estimated weight]/actual weight)” and averaged across the three trials. A score closer to 1 indicated a higher accuracy of heaviness perception.

### Heartbeat counting task

The HCT was administered according to Schandry’s method [26]. For this purpose, the heart rate was objectively recorded using a pulse oximeter (227AKBZX00031000, C. I. Medical Co., Ltd.). At the same time, the participants silently counted their heartbeats over three trials of different durations. The order of the intervals (25, 35, and 45 s) was randomized. They were instructed to keep their eyes closed and avoid manually measuring their pulses. After each trial, the participants reported their heartbeat counts. They were told to respond with “zero” when they did not perceive their heartbeat.

The HCT score was calculated using a similar approach to that of the absolute error scores: “1 – ([actual heartbeat – participant’s reported heartbeat]/actual heartbeat).” The scores were averaged across the three trials. A score closer to 1 indicates higher accuracy of the HCT.

After completing the HCT, participants were also asked about their subjective impressions. Specifically, they were asked about the extent to which they guessed during reporting and how much they relied on their knowledge of typical resting heart rates, estimated elapsed time, and utilized pulse sensation from the pulse oximeter attachment site [27]. These were measured using 100-mm visual analog scales (VAS), with labels ranging from “did not rely or guess at all” to “relied or guessed completely.” The mean VAS scores were calculated for the three trials.

### Self-report questionnaires

#### Body Perception Questionnaire-Body Awareness very Short form

The BPQ-VSF [18] was administered to measure interoceptive sensibility. It comprises 12 items selected from the original 26-item BPQ-BA, scored on a 5-point scale. Higher scores indicate greater interoceptive awareness.

#### 20-Item Toronto Alexithymia Scale (TAS-20)

The TAS-20 [12] was administered to assess alexithymia. Each item is rated on a 5-point scale, and the subscale (i.e., DIF, DDF, and EOT) and total scores were calculated. A higher total score indicates a greater alexithymia tendency.

#### Shitsu-Taikan-Sho-Scale (STSS)

The STSS [10] was administered to assess alexisomia. It consists of 23 items, including three subscales (i.e., DIB, OA, and LHM). Each item is rated on a 5-point scale, and the subscale and total scores were calculated. A higher total score indicates a greater alexisomia tendency.

### Data analysis

Data were analyzed using SPSS for Windows (version 30.0.0.0; IBM). The Shapiro–Wilk test was used to examine the normality of the heaviness perception test scores, as well as all other outcome measures. Pearson product-moment correlation coefficients were calculated to comprehensively examine the relationships among the heaviness perception test, HCT, BPQ-VSF, TAS-20, and STSS scores. Correlation coefficients were also calculated between the scores of the heaviness perception test and each subscale of the TAS-20 and STSS. Partial correlation analyses between the heaviness perception test and the HCT scores were performed, controlling for subjective impressions after the HCT (i.e., guessing during reporting, reliance on knowledge of typical resting heart rate, estimation of elapsed time, and use of pulse sensations from the pulse oximeter attachment site). The significance level was set at 0.05.

## Results

**Fig. 2:**
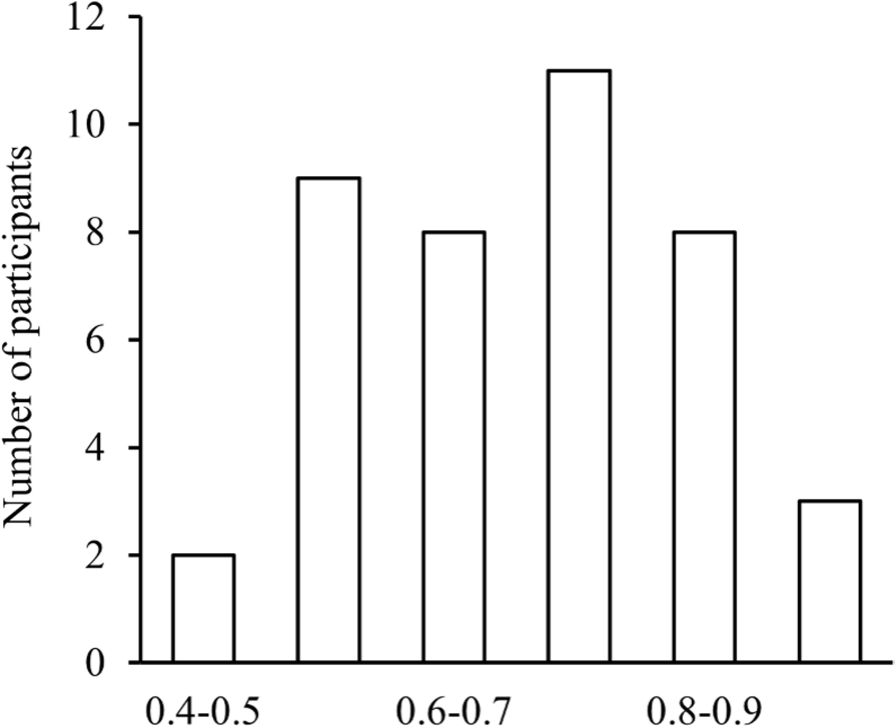
Distribution of the heaviness perception scores

**Fig. 3:**
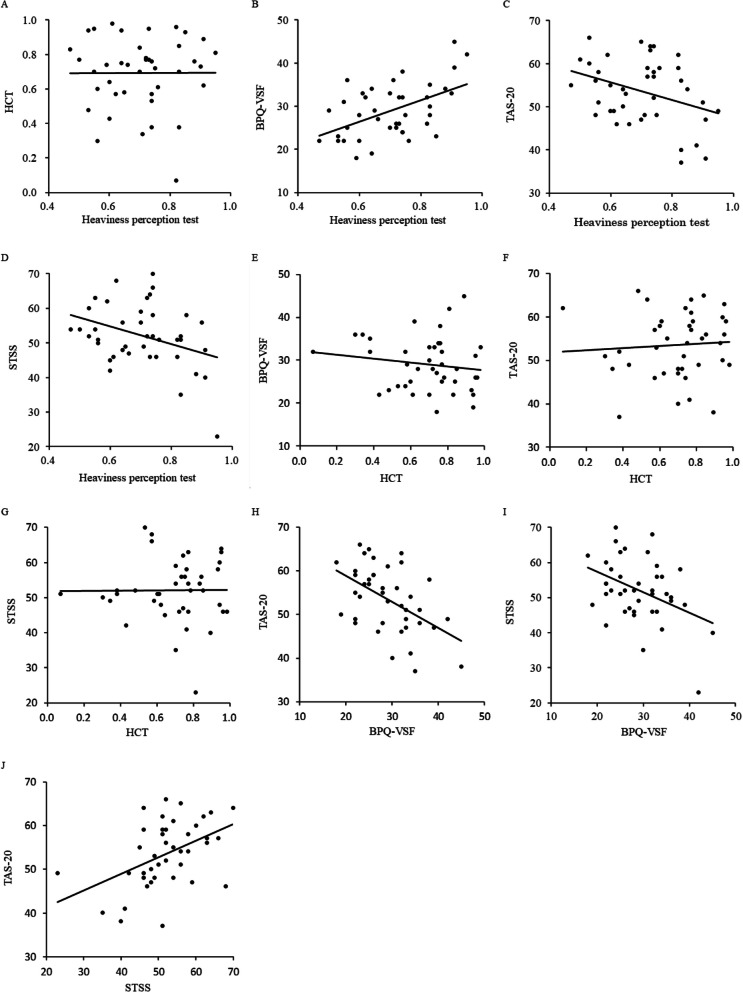
Correlations among the heaviness perception test, HCT, BPQ-VSF, TAS-20, and STSS scores. Panels **A**–**J **Show the correlations among the scores of heaviness perception test, HCT, BPQ-VSF, TAS-20, and STSS. Significant correlations were observed in Panels **B**, **C**, **D**, **H**, **I**, and **J**. No significant correlations were observed in Panels **A**, **E**–**G**. Abbreviations. HCT: Heartbeat Counting Task; BPQ-VSF: Body Perception Questionnaire–Body Awareness Very Short Form; TAS-20: 20-item Toronto Alexithymia Scale; STSS: Shitsu-taikan-sho Scale

The distribution of the heaviness perception scores is shown in Fig. 2. The Shapiro–Wilk test confirmed that the heaviness perception test scores were normally distributed (*p* = 0.37). Similarly, the HCT and BPQ-VSF scores, as well as the TAS-20 and STSS scores (total and subscales), were normally distributed (*ps* > 0.17). Descriptive statistics for the heaviness perception test, HCT, BPQ-VSF, TAS-20, and STSS, along with their correlation coefficients, are presented in Table 1. The correlations among the heaviness perception test, HCT, BPQ-VSF, TAS-20, and STSS scores are shown in Fig. 3. The heaviness perception test score demonstrated a significant positive correlation with the BPQ-VSF (*r* = 0.504, *p* < 0.01; Table 1, Fig. 3B) and significant negative correlations with the TAS-20 (*r* = –0.342, *p* < 0.05) and STSS (*r* = –0.353, *p* < 0.05) total scores, as illustrated in Table 1 and Fig. 3B–D. Table 2 shows the descriptive statistics and correlation coefficients among the heaviness perception test score, TAS-20 subscale scores, and STSS subscale scores. A significant negative correlation was found between the heaviness perception test score and the DIF subscale scores of the TAS-20 (*r* = –0.367, *p* < 0.05). Furthermore, a significant negative correlation was found between the perception test score and the DIB subscale score of the STSS (*r* = –0.369, *p* < 0.05).

**Table 1:** Correlations among the heaviness perception test, HCT, BPQ-VSF, TAS-20, and STSS scores

	Mean	Standard deviatio	1	2	3	4	5
1. Heaviness perception test	0.70	0.13	1				
2. HCT	0.69	0.21	.005	1			
3. BPQ- VSF	29.02	6.22	.504 **	-.149	1		
4. TAS-20	53.51	7.49	-.342 *	.068	-.500 **	1	
5. STSS	52.05	9.05	-.353 *	.009	-.401 **	.459 **	1

*Abbreviations*. *HCT* Heartbeat Counting Task, *BPQ-VSF* Body Perception Questionnaire–Body Awareness Very Short Form, *TAS-20* 20-item Toronto Alexithymia Scale, *STSS* Shitsu-taikan-sho Scale

^*^Denotes significance at *p* <.05

^**^Denotes significance at *p* <.01

**Table 2:** Correlations among the heaviness perception test, the TAS-20 subscale scores, and the STSS subscale scores

		Mean	Standard deviation	*r*	*p*
TAS-20	DIF	16.20	4.99	-.367 *	.020
	DDF	15.43	3.32	-.182	.261
	EOT	21.63	3.99	-.054	.739
STSS	DIB	18.90	5.73	-.369 *	.019
	OA	14.88	3.74	-.207	.199
	LHM	18.43	4.41	-.073	.656

*Abbreviations*. *TAS-20* 20-item Toronto Alexithymia Scale, *DIF* Difficulty Identifying Feelings, *DDF* Difficulty describing feelings to others, *EOT* Externally oriented thinking, *STSS* Shitsu-taikan-sho Scale, *DIB* Difficulty of identifying bodily feelings, *OA* Over–adaptation, *LHM* Lack of health management based on bodily feelings

^*^Denotes significance at *p* <.05

The BPQ-VSF score also showed significant negative correlations with the TAS-20 (*r* = –0.500, *p* < 0.01) and STSS (*r* = –0.401, *p* < 0.01) total scores, as shown in Table 1 and Fig. 3H–I. Moreover, a significant positive correlation was found between the TAS-20 and STSS total scores (*r* = 0.459, *p* < 0.01; Table 1, Fig. 3J).

However, no significant correlations were observed between the HCT score and the BPQ-VSF score (*r* = –0.149, *p* = 0.353), TAS-20 total score (*r* = 0.068, *p* = 0.670), or STSS total score (*r* = 0.009, *p* = 0.956), as illustrated in Table 1 and Fig. 3E–3G.

The relationship between the heaviness perception test and the HCT score is illustrated in Fig. 3A. Table 3 shows the means and standard deviations of participants’ subjective impressions after the HCT (i.e., VAS scores for guessing during reporting, reliance on knowledge of typical resting heart rate, estimation of elapsed time, and use of pulse sensations from the pulse oximeter attachment site). A partial correlation analysis controlling for these subjective impressions revealed no significant correlation between the heaviness perception test and the HCT score (*r* = –0.029, *p* = 0.866).

**Table 3:** Subjective impressions after the HCT (VAS scores)

	Mean	Standard deviation
Guessing during reporting	46.2	27.2
Knowledge of resting heart rate	16.8	24.5
Estimation of elapsed time	19.7	29.0
Pulse sensation from the pulse oximeter attachment site	31.6	31.5

## Discussion

In this study, we investigated the relationship between the heaviness perception test and the BPQ-VSF to determine the relationship between their assessments of interoceptive accuracy and sensibility. In addition, we examined possible associations of the heaviness perception test score with the TAS-20 (total alexithymia score and subscale scores, DIF, DDF, and EOT) and STSS (total alexisomia score and subscale scores, DIB, OA, and LHM) because interoceptive dysfunction has been reported to occur in alexithymia and alexisomia. Moreover, we examined the relationships among the HCT, which has been used as an index of interoceptive accuracy, BPQ-VSF, TAS-20, and STSS. The results showed that the heaviness perception test score had a significant positive correlation with BPQ-VSF score and a negative correlation with the TAS-20 and STSS total scores. In particular, the heaviness perception test showed a significant negative correlation with the DIF subscale of the TAS-20 and the DIB subscale of the STSS. On the other hand, the HCT score was not correlated with the BPQ-VSF, TAS-20, or STSS scores. Moreover, no correlation was detected between the heaviness perception test and HCT scores when the analysis controlled for factors that influence the HCT.

The results of the heaviness perception test followed a normal distribution in this study, suggesting that the test adequately captured the participants' perceptions of heaviness, which provides support for the validity of the method. The heaviness perception test results in this study were consistent with those of Murphy et al. [13]. In their experiment, the baseline bucket contained rice and the experimenter poured rice into the test bucket as a comparative stimulus. The amount of rice placed in the bucket depended on the experimenter’s manipulation, and the speed at which the rice was added may have influenced the participants’ heaviness evaluations. In contrast, we devised a method that supplied water at a constant speed as our comparative stimulus. Despite the smaller sample size in our study, the data maintained a normal distribution. Furthermore, a significant correlation was found between the test scores that reflected sensibility to interoception (BPQ-VSF). Additionally, the correlation between the heaviness perception test and the TAS-20 was consistent with the findings of Murphy et al. [13]. These results suggest that the heaviness perception test used in this study is a valid indicator of interoceptive accuracy.

The positive correlation between the heaviness perception test and BPQ-VSF scores suggests an association between the assessment of interoception in different domains. This study is a pilot examination of the heaviness perception test to assess interoception. Heaviness perception is generally considered part of proprioception [29], and proprioception is considered an aspect of interoception [1, 2, 13, 15, 31, 32]. In addition, muscle tension when lifting an object can trigger the exercise pressor reflex, which is considered to be part of the afferent pathway of interoception [13, 37]. Based on this theoretical background and the characteristics of the measurement method used in this study (i.e., an objectively quantifiable approach to individual differences in behavioral performance [13, 25]), the heaviness perception test can be considered as an assessment of interoception accuracy. The BPQ-VSF was originally a self-report measure of the awareness of bodily sensations [18], but it is used as an index to evaluate interoception sensibility [15, 19–23]. Previous studies have highlighted inconsistencies in the assessments of interoceptive accuracy and sensibility, and the discrepancy between the two domains has been related to the symptoms of mental and physical illnesses [16, 25]. As mentioned above, there are several issues with the assessment of interoceptive accuracy when using the HCT. In fact, this study found no correlation between the HCT and BPQ-VSF scores, but showed a correlation between the heaviness perception test and BPQ-VSF scores, suggesting that the heaviness perception test has potential as a method for evaluating interoception. Further research is needed to determine whether the heaviness perception test can be used to evaluate interoceptive accuracy and sensibility. Incorporating questionnaire measures and considering various perceived interoceptive modalities [13] would create a more comprehensive approach.

In this study, negative correlations were found between the heaviness perception test and the TAS-20 and STSS scores. This suggests that this method would be useful for the evaluation of interoceptive dysfunction associated with alexithymia and alexisomia. Specifically, the heaviness perception test score had a significant negative correlation with the TAS-20 total and DIF subscale scores, indicating that individuals with a lower ability to perceive and recognize heaviness tended to have difficulty identifying their emotions. The negative correlation of the TAS-20 total and heaviness perception test scores is consistent with previous research [13]. No research has examined the relationship between the heaviness perception test and STSS scores. However, the relationship between the heaviness perception test and STSS total scores and the DIB subscale scores showed that individuals who were unable to accurately perceive the sensation of weight tended to be unaware of bodily sensations.

In addition to alexithymia and alexisomia, children with developmental disorders (such as autism spectrum disorder, developmental coordination disorder, and attention deficit hyperactivity disorder) may have interoceptive dysfunction [38, 39]. The heaviness perception test shown in this study can be performed relatively easily with children, in contrast with the HCT which would be difficult to use with them. A procedure based on the perception of heaviness may be useful for screening for developmental disorders and providing early support. Basic studies on the validity of evaluating interoception when using the heaviness perception test will be necessary that include a larger sample size and that include children.

This study had several limitations. First, the sample consisted of only healthy young people and the number of participants was small. A more diverse and larger sample size that includes children will be needed in future studies. Second, our method involved pouring water at a constant speed as a comparison stimulus in the heaviness perception test. Although the participants were not informed of these methods in detail, it is necessary to examine the possibility of the predictive coding model hypothesis [40–42] in our results: when applied to the heaviness perception test, interoception (heaviness perception) may have been miscalculated because of prediction error between the brain and the actual input from the upper limb holding the bucket when water is being poured into it, updating of the prediction model, or changing the state of the upper limb (e.g., muscle tension) to adjust for the input. Further research will be necessary to determine the influence of the predictive coding model on the results of the heaviness perception test.

## Conclusion

Our study revealed significant correlations between the heaviness perception test and several established interoceptive indices. These findings support the feasibility of using the heaviness perception test as a simple and practical method for assessing interoception. Future studies should explore its applicability to children and clarify its neurobiological underpinnings to strengthen its validity as an interoceptive assessment tool.

## Data Availability

The datasets generated in this study are available from the corresponding author upon reasonable request from the consenting participants.
